# Supplementary material for: Repeated Evolution of Asexuality Involves Convergent Gene Expression Changes
Source: Mol Biol Evol. 2018 Nov 16;36(2):350–64. doi: 10.1093/molbev/msy217 (PMC6404633; doi:10.1093/molbev/msy217)
Supplement: Supplementary Data [file msy217_supp.zip › Tim_Convergent_changes_in_GE_asexuality_v9b_supp.pdf]

Supplementary Figures

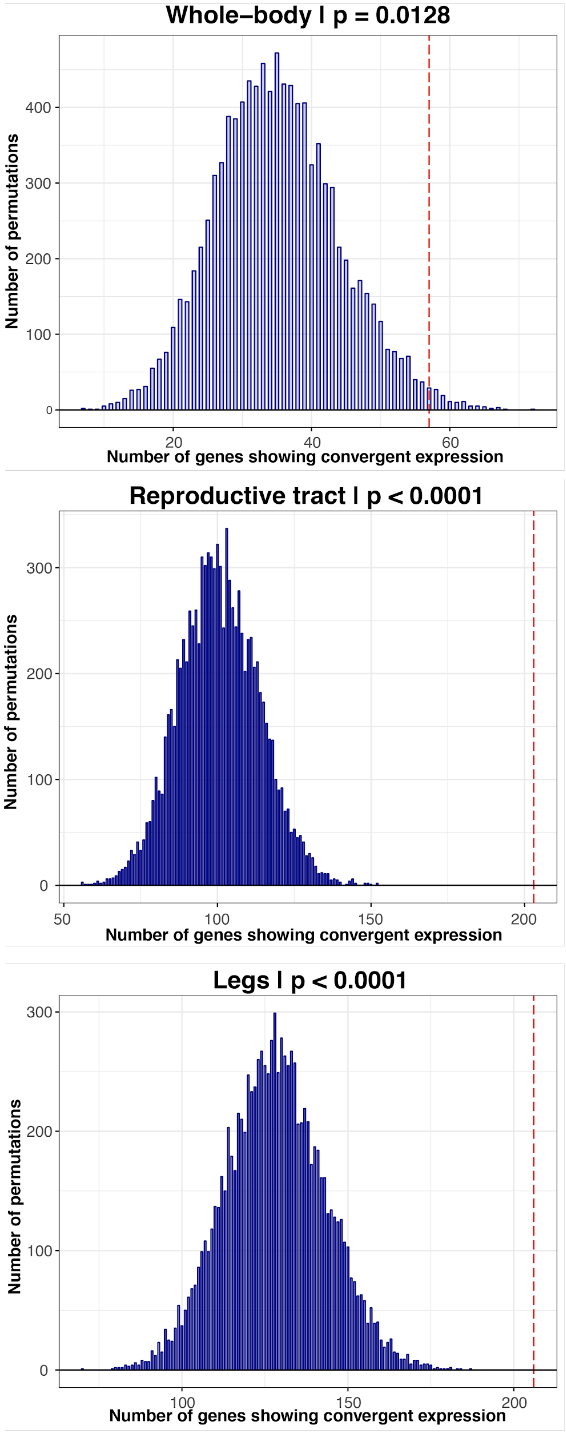

Supplementary Figure 1 | Number of genes expected to show a convergent expression pattern by chance (assessed by assigning reproductive mode randomly within species pairs for each gene for 10,000 permuted datasets). The observed number of convergent genes is indicated by the red dashed line. P-values refer to the probability of observing a number of convergent genes greater-than or equal-to the observed value.

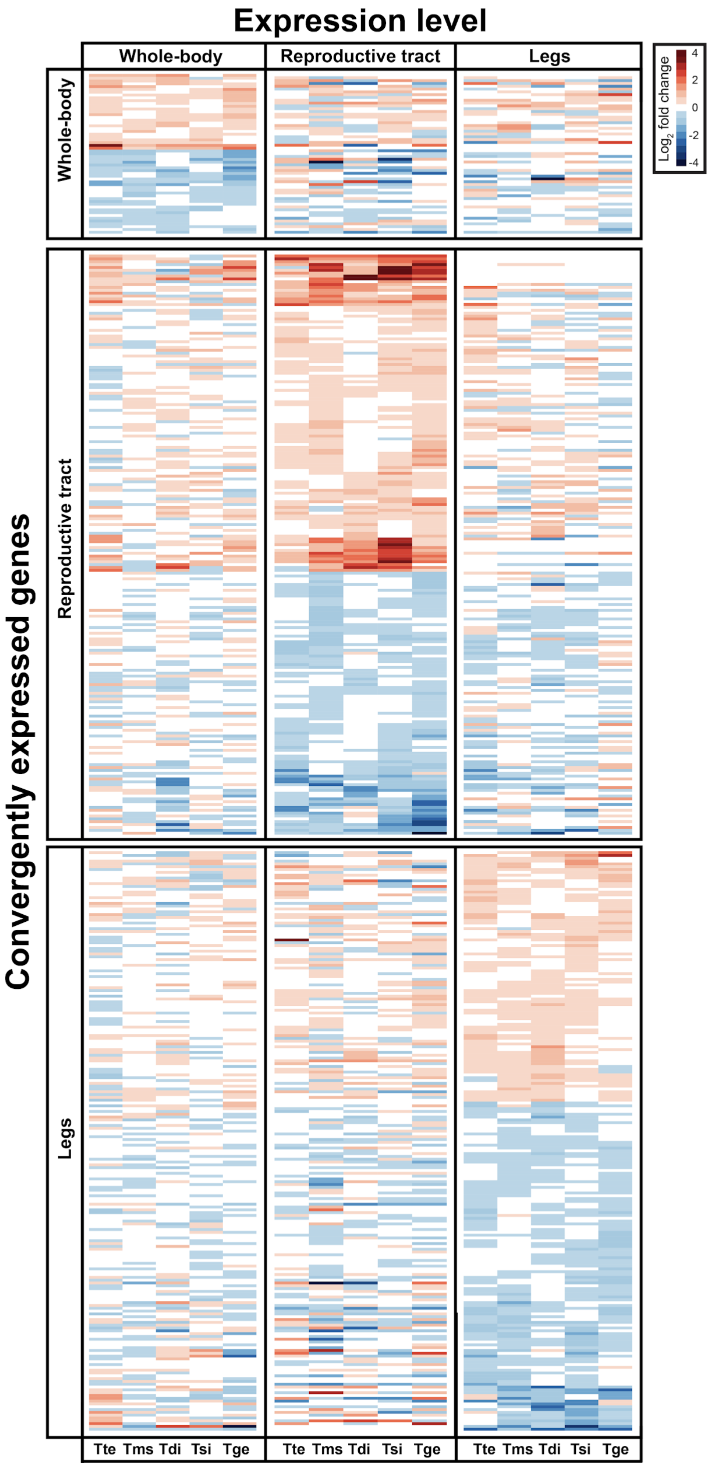

Supplementary Figure 2 | Convergently changing genes are largely tissue-specific. Heatmaps of genes showing convergent gene expression changes between sexual and asexual females for whole-bodies, reproductive tract, and legs, including their expression in other tissues. Species names are abbreviated as follows: Tte = *T. tahoe*, Tms = *T. monikensis*, Tdi = *T. douglasi*, Tsi = *T. shepardi*, and Tge = *T. genevievae*.

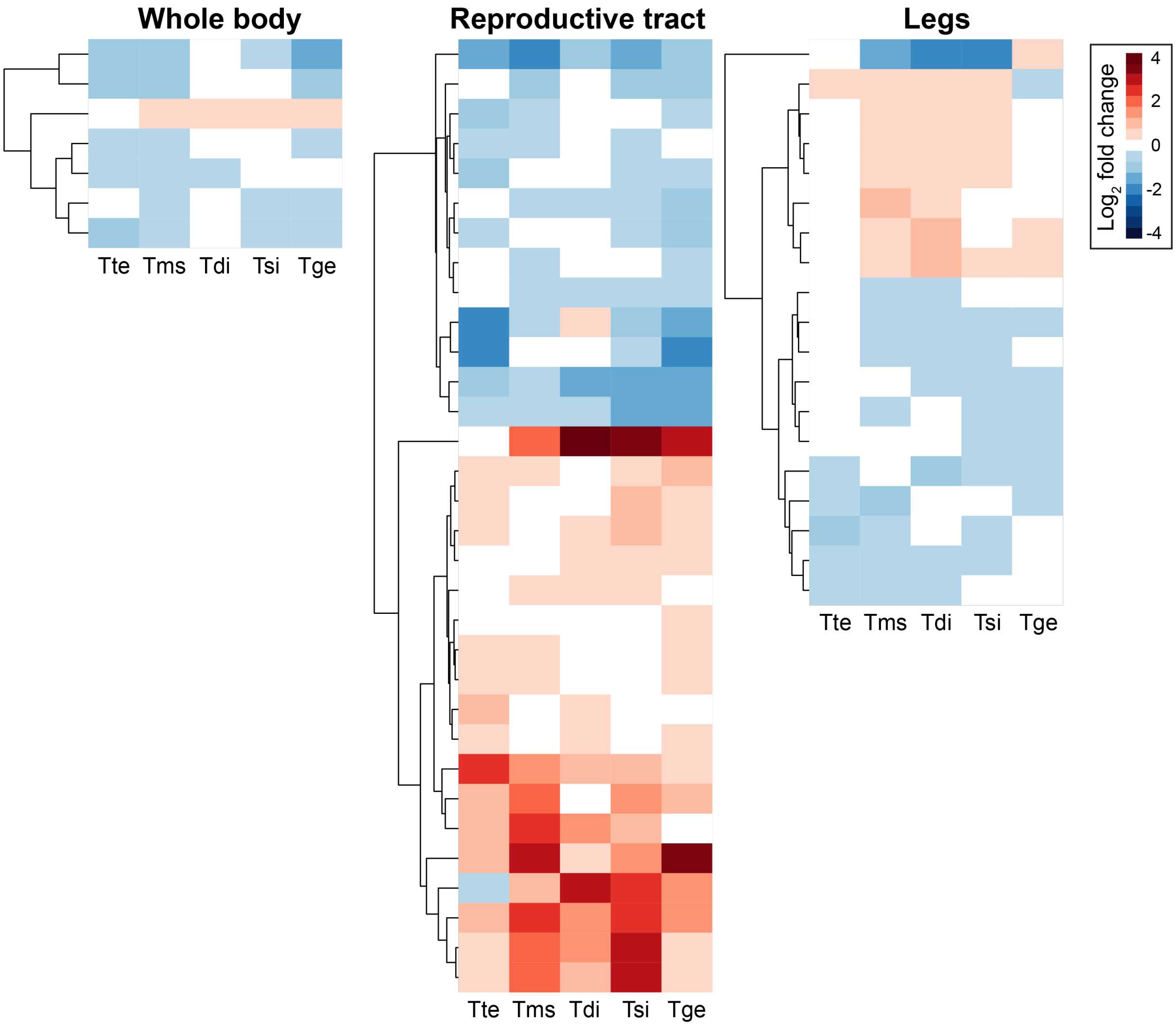

Supplementary Figure 3 | Heatmaps of expression changes between sexual and asexual females for whole-bodies, reproductive tract, and legs for genes with a better fit to the multi-optima model than to the two-state adaptive optimal model. Species names are abbreviated as follows: Tte = *T. tahoe*, Tms = *T. monikensis*, Tdi = *T. douglasi*, Tsi = *T. shepardi*, and Tge = *T. genevievae*.

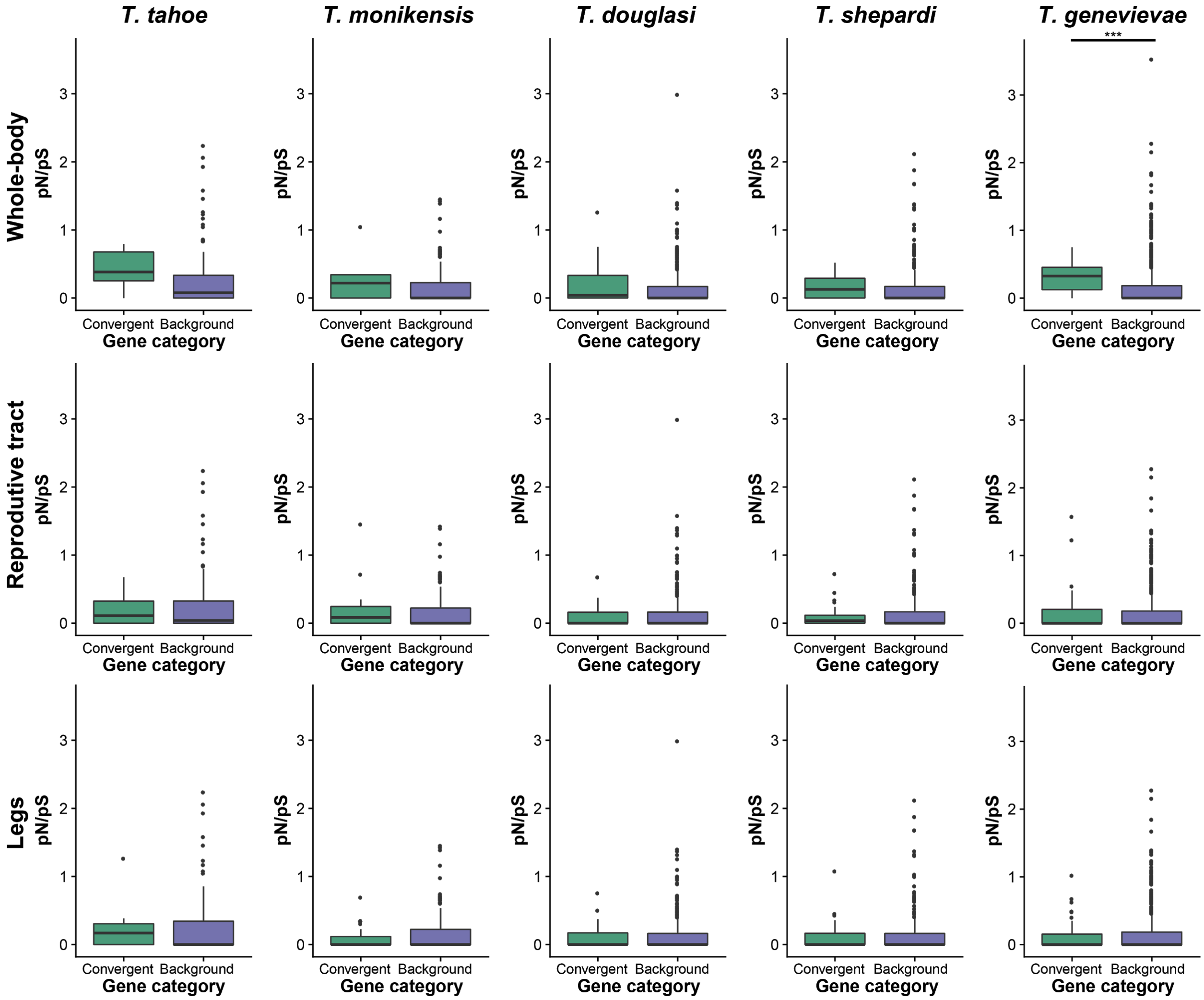

Supplementary Figure 4 | pN/pS ratios for convergently expressed genes versus all other genes expressed in that tissue for whole-bodies, reproductive tracts and legs for each asexual species. Significance is indicated by asterisks (*** < 0.001) from a Wilcoxon test.

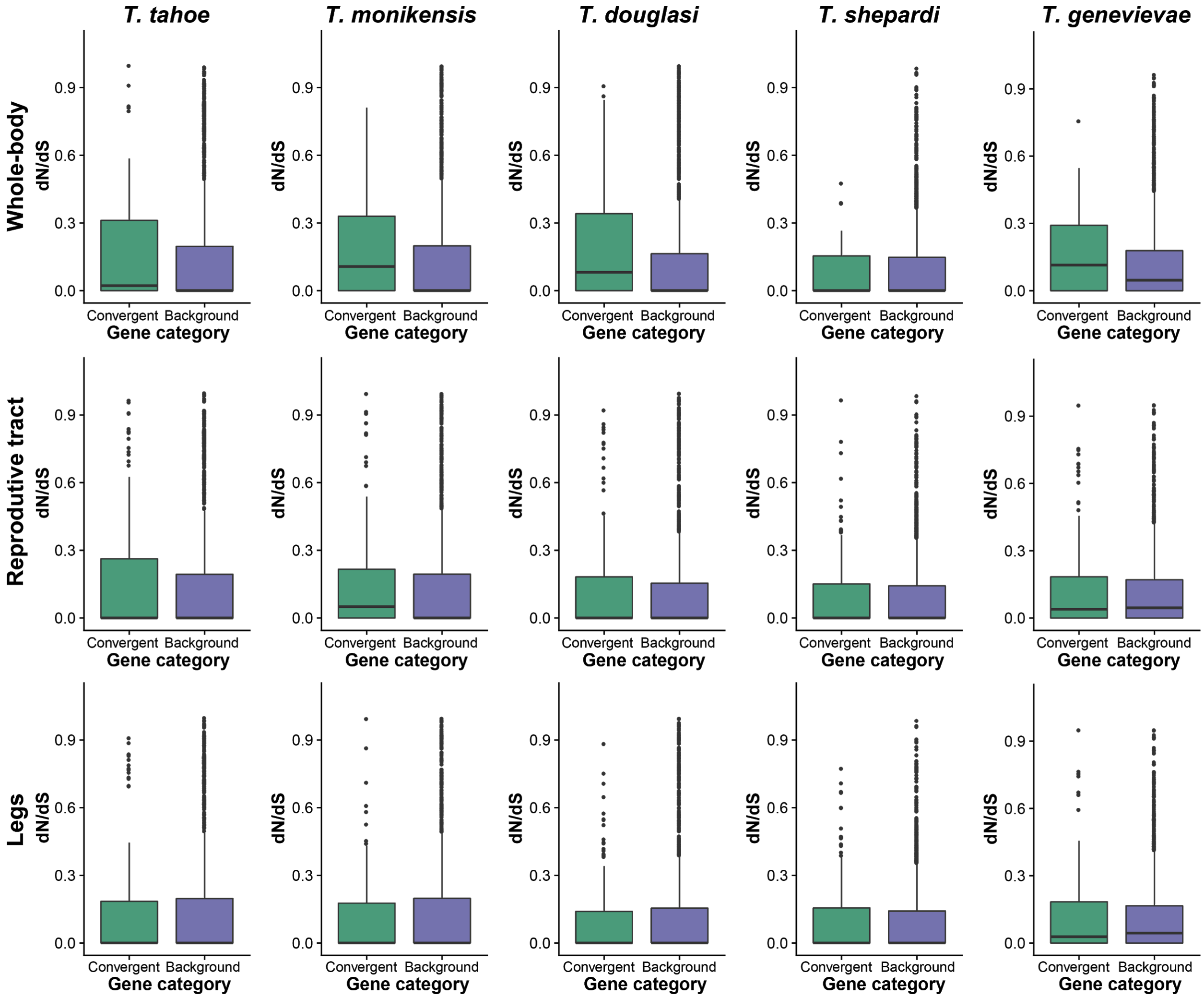

Supplementary Figure 5 | dN/dS ratios for convergently expressed genes versus all other genes expressed in that tissue for whole-bodies, reproductive tract and legs for each asexual species. No comparisons were significantly different (Wilcoxon test, $p > 0.05$).

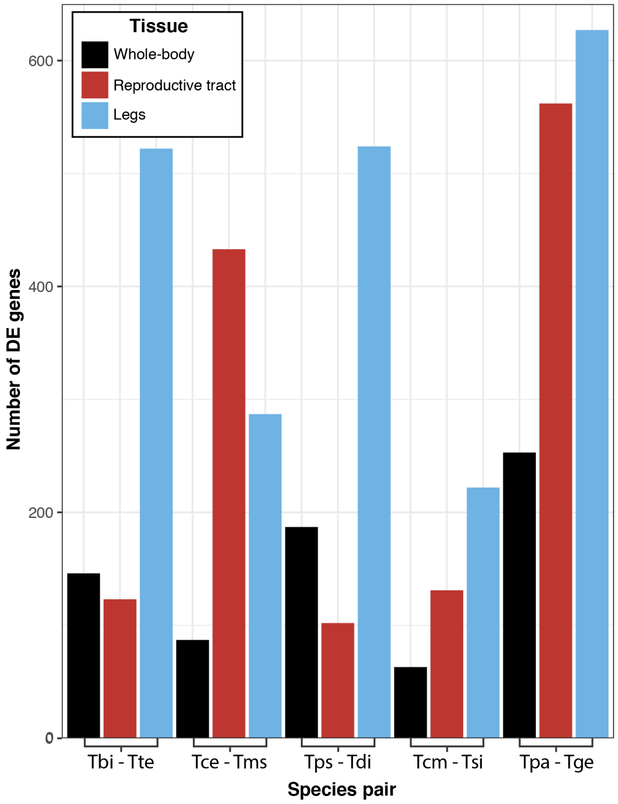

Supplementary Figure 6 | Number of DE genes (FDR < 0.05) between sexual and asexual females for each species-pair for whole-body, reproductive tract, and legs for the 10 species orthologs. Species names are abbreviated as follows: Tbi = *T. bartmani*, Tce = *T. cristinae*, Tps = *T. poppensis*, Tcm = *T. californicum*, Tpa = *T. podura*, Tte = *T. tahoe*, Tms = *T. monikensis*, Tdi = *T. douglasi*, Tsi = *T. shepardi*, and Tge = *T. genevievae*.

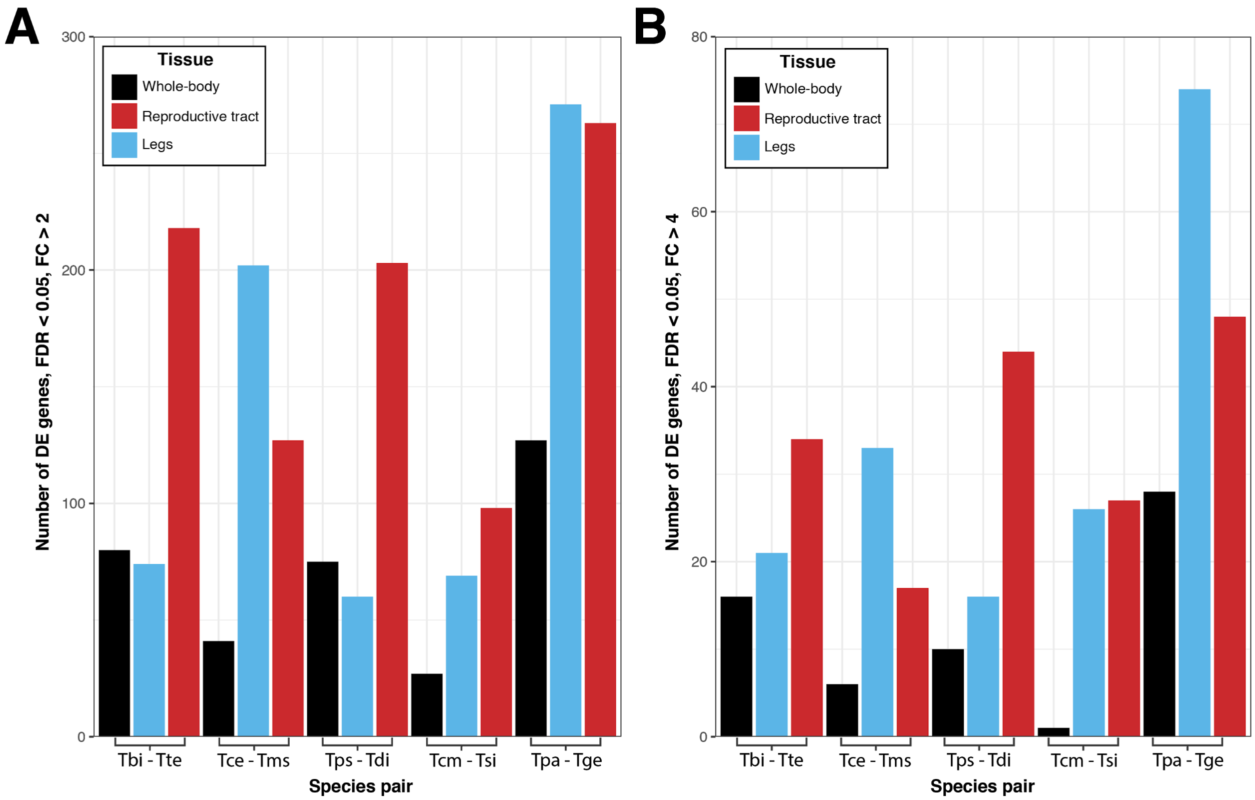

Supplementary Figure 7 | Number of DE genes (FDR < 0.05) between sexual and asexual females for each species-pair for whole-body, reproductive tract, and legs for the 10 species orthologs with different fold-change thresholds. A. Number of significantly DE genes with a fold-change greater than 2, B Number of significantly DE genes with a fold-change greater than 4. Species names are abbreviated as follows: Tbi = *T. bartmani*, Tce = *T. cristinae*, Tps = *T. poppensis*, Tcm = *T. californicum*, Tpa = *T. podura*, Tte = *T. tahoe*, Tms = *T. monikensis*, Tdi = *T. douglasi*, Tsi = *T. shepardi*, and Tge = *T. genevievae*.

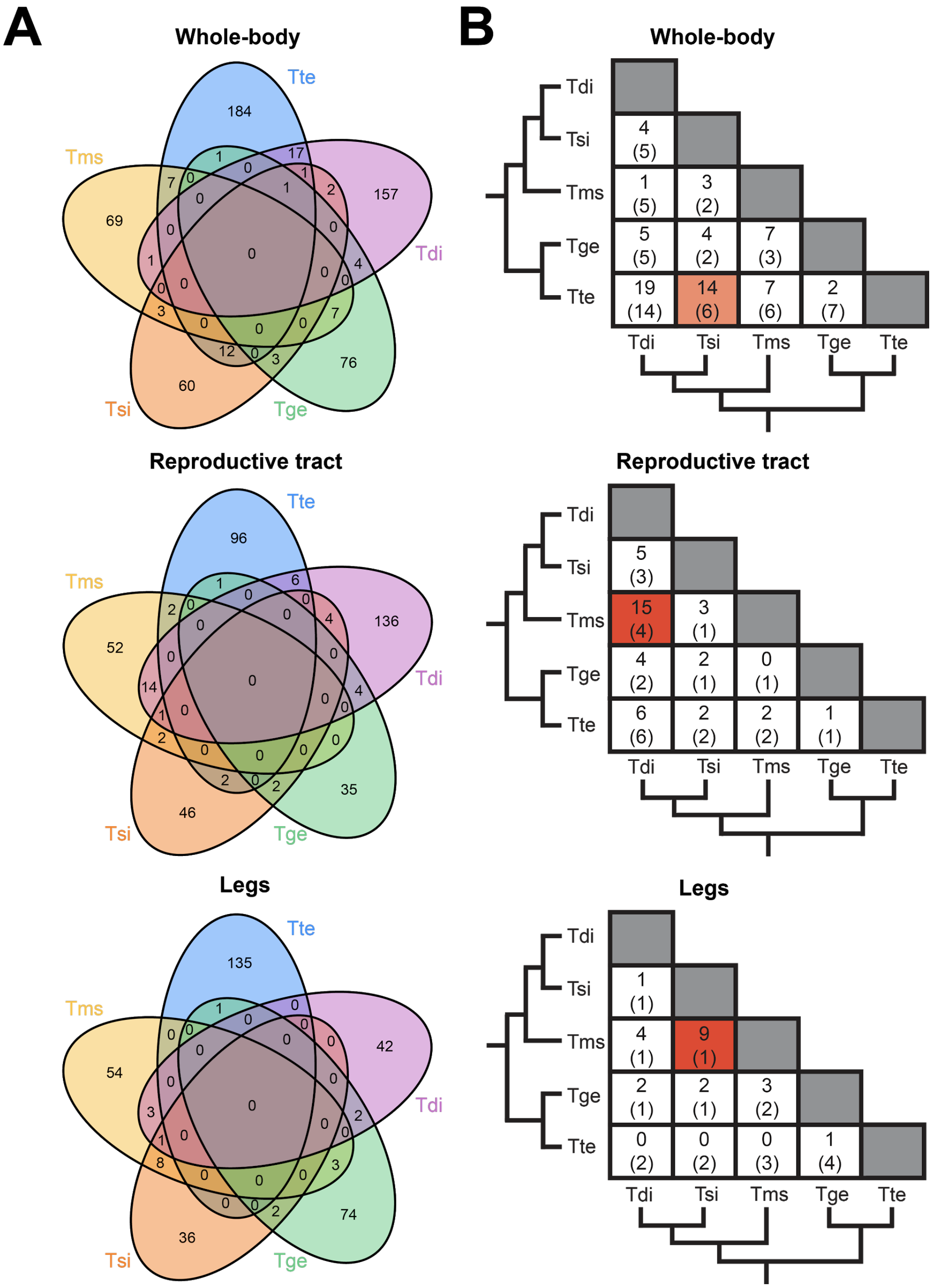

Supplementary Figure 8 | A. Venn-diagrams showing the number of enriched GO-terms between sexual and asexual females that are shared among species-pairs for whole-body, reproductive tracts, and legs for 10 species orthologs (FDR < 0.05). B. Matrices showing pairwise overlap of enriched GO-terms between sexual and asexual females with the number of GO terms expected by chance given in parentheses. Colours represent a significantly greater overlap than expected by chance (red, FDR < 0.001, orange < 0.05). The phylogeny shows the relationships between asexual species (from Schwander et al (2011)). Species names are abbreviated as follows: Tte = *T. tahoe*, Tms = *T. monikensis*, Tdi = *T. douglasi*, Tsi = *T. shepardi*, and Tge = *T. genevievae*.

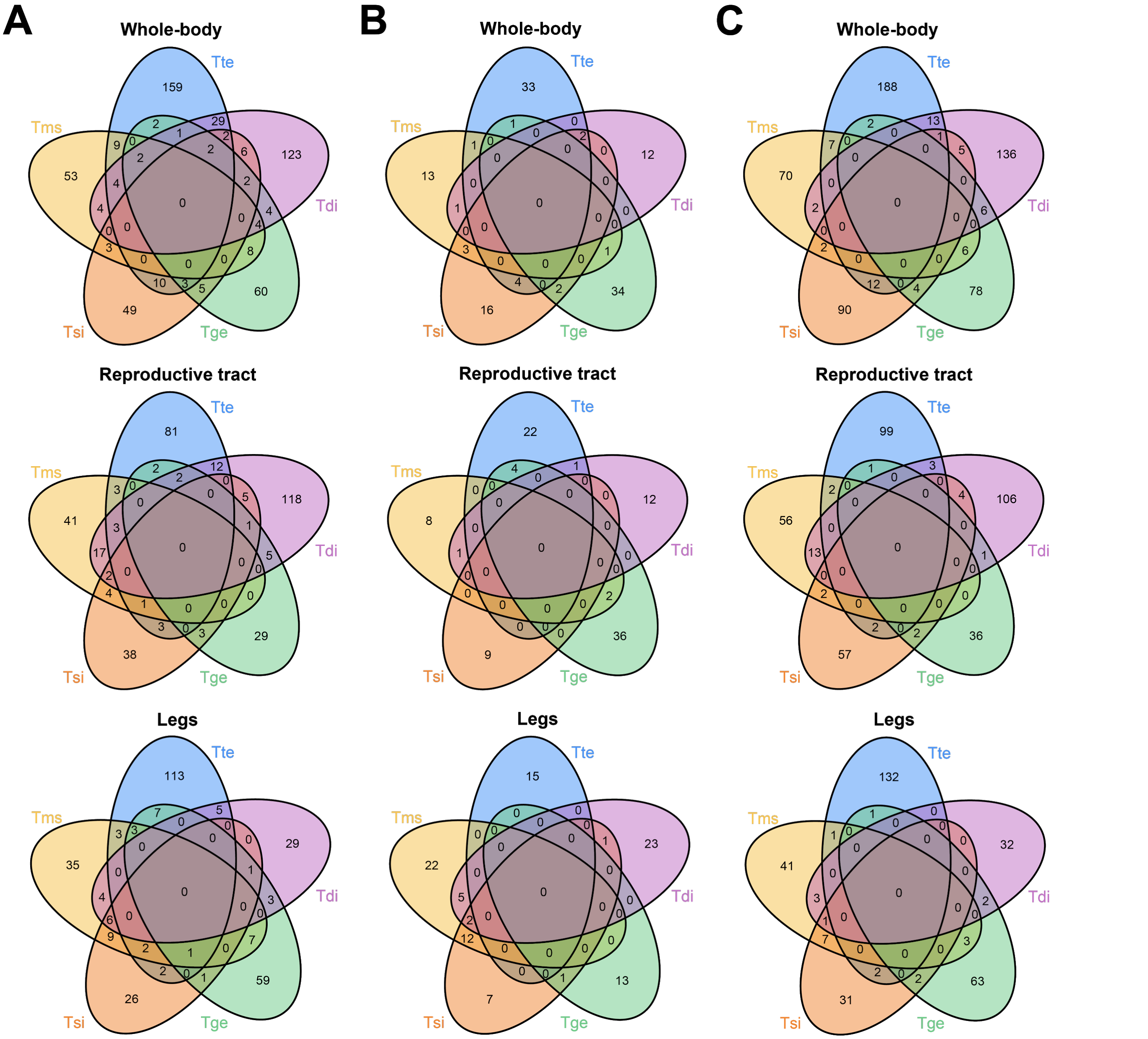

Supplementary Figure 9 | Venn-diagrams showing the number of enriched GO-terms ($p < 0.05$) for differences between sexual and asexual females that are shared among species-pairs in the whole-body, reproductive tract, and legs when: **A** GO terms were first clustered together based on parent or child terms, **B** only NCBI's nr annotation was used, **C** only the *T. bartmani* annotation. Species names are abbreviated as follows: Tte = *T. tahoe*, Tms = *T. monikensis*, Tdi = *T. douglasi*, Tsi = *T. shepardi*, and Tge = *T. genevievae*.

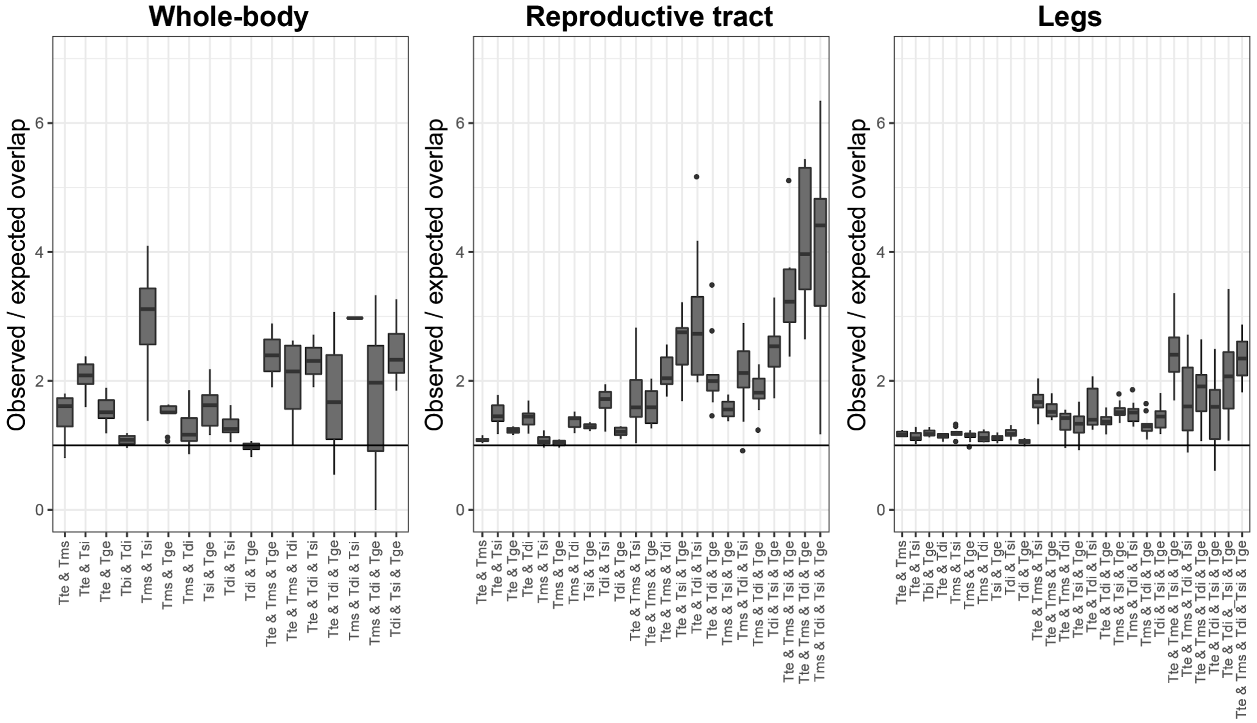

Supplementary Figure 10 | Ratio of observed to expected amount of overlap for genes when reads were mapped to the whole transcriptome for each species. Note boxes are only shown when the expected overlap was >= 1. Species names are abbreviated as follows: Tte = *T. tahoe*, Tms = *T. monikensis*, Tdi = *T. douglasi*, Tsi = *T. shepardi*, and Tge = *T. genevievae*.

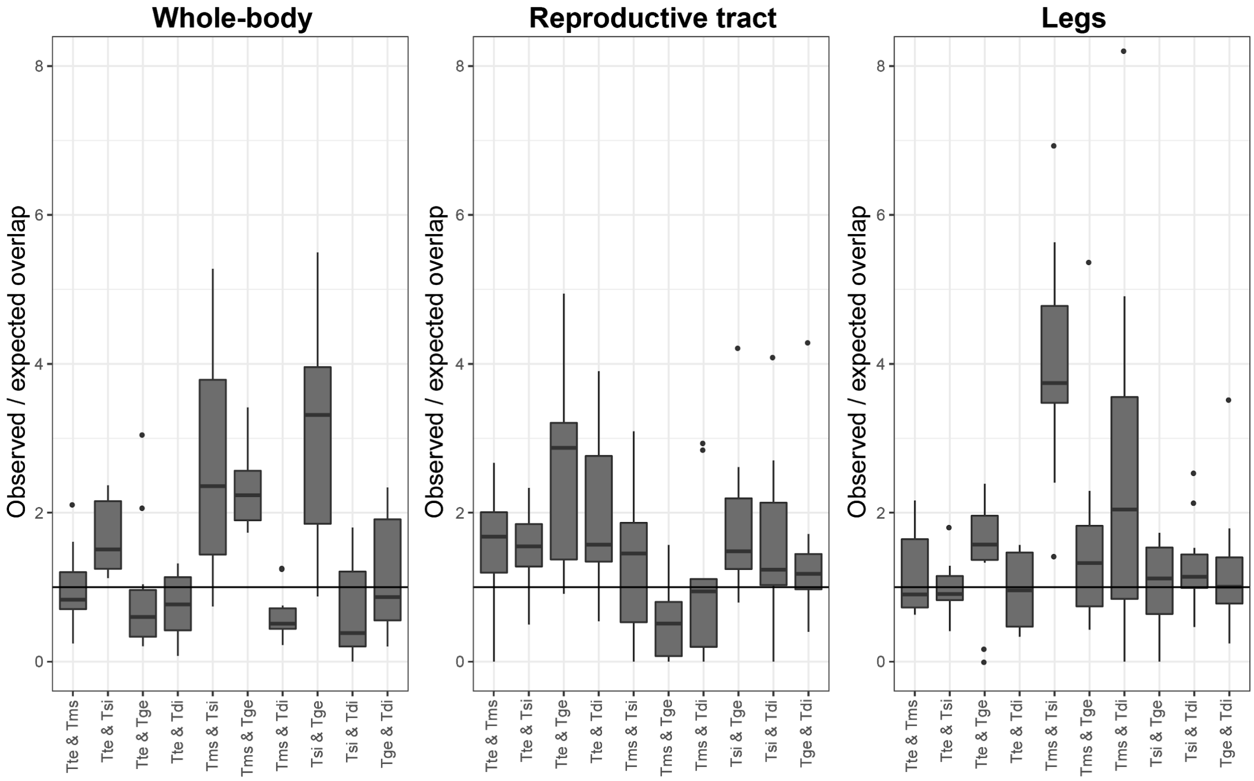

Supplementary Figure 11 | Ratio of observed to expected amount of overlap of enriched GO-terms when reads were mapped to the whole transcriptome for each species. Note boxes are only shown when the expected overlap was >= 1. Species names are abbreviated as follows: Tte = *T. tahoe*, Tms = *T. monikensis*, Tdi = *T. douglasi*, Tsi = *T. shepardi*, and Tge = *T. genevievae*.

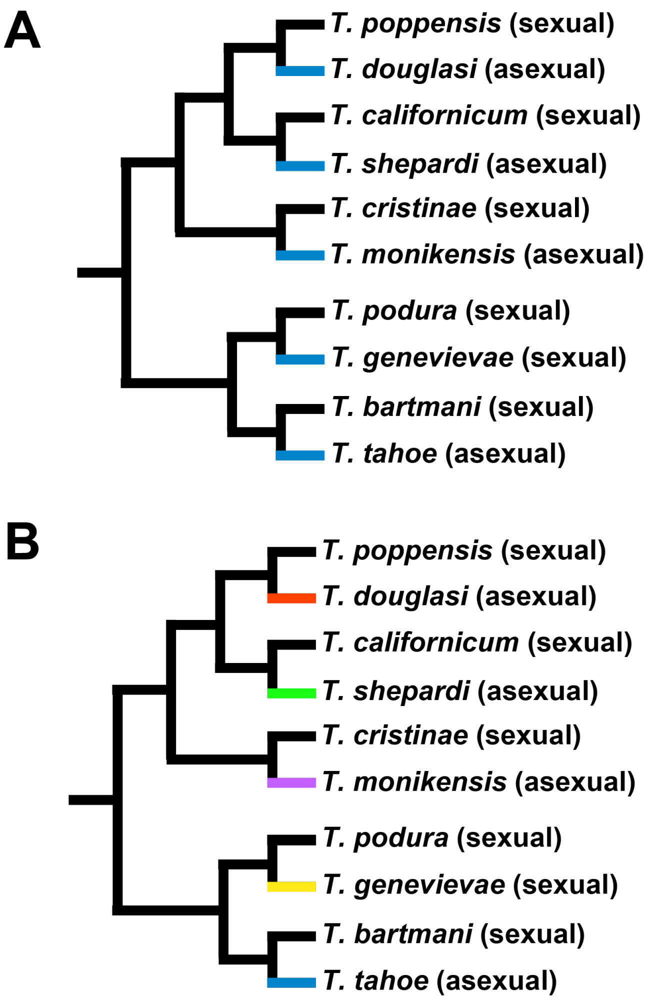

Supplementary Figure 12 | Different selective scenarios tested using the OU framework. **A.** a two-state adaptive-optima model where asexual species (blue) had a different adaptive-optima than sexual species (black). **B.** Multi-asexual optima model where sexual species (black) and each asexual species had different optima (colored branches).
